# Diversification of Colonization Factors in a Multidrug-Resistant Escherichia coli Lineage Evolving under Negative Frequency-Dependent Selection

**DOI:** 10.1128/mBio.00644-19

**Published:** 2019-04-23

**Authors:** Alan McNally, Teemu Kallonen, Christopher Connor, Khalil Abudahab, David M. Aanensen, Carolyne Horner, Sharon J. Peacock, Julian Parkhill, Nicholas J. Croucher, Jukka Corander

**Affiliations:** a Institute of Microbiology and Infection, University of Birmingham, Birmingham, United Kingdom; b Infection Genomics, Wellcome Sanger Institute, Cambridge, United Kingdom; c Department of Biostatistics, University of Oslo, Oslo, Norway; d British Society of Antimicrobial Chemotherapy, Birmingham, United Kingdom; e Department of Medicine, University of Cambridge, Cambridge, United Kingdom; f London School of Hygiene and Tropical Medicine, London, United Kingdom; g Faculty of Medicine, School of Public Health, Imperial College, London, United Kingdom; h Department of Mathematics and Statistics, University of Helsinki, Helsinki, Finland; University of British Columbia; University of Montreal; University of Oxford

**Keywords:** AMR, *Escherichia coli*, evolutionary genomics, negative frequency-dependent selection

## Abstract

Escherichia coli is a major cause of bloodstream and urinary tract infections globally. The wide dissemination of multidrug-resistant (MDR) strains of extraintestinal pathogenic E. coli (ExPEC) poses a rapidly increasing public health burden due to narrowed treatment options and increased risk of failure to clear an infection. Here, we present a detailed population genomic analysis of the ExPEC ST131 clone, in which we seek explanations for its success as an emerging pathogenic strain beyond the acquisition of antimicrobial resistance (AMR) genes. We show evidence for evolution toward separate ecological niches for the main clades of ST131 and differential evolution of anaerobic metabolism, key colonization, and virulence factors. We further demonstrate that negative frequency-dependent selection acting across accessory loci is a major mechanism that has shaped the population evolution of this pathogen.

## Abstract (precis)

Infections with multidrug-resistant (MDR) strains of Escherichia coli are a significant global public health concern. To combat these pathogens, we need a deeper understanding of how they evolved from their background populations. By understanding the processes that underpin their emergence, we can design new strategies to limit evolution of new clones and combat existing clones. By combining population genomics with modelling approaches, we show that dominant MDR clones of E. coli are under the influence of negative frequency-dependent selection, preventing them from rising to fixation in a population. Furthermore, we show that this selection acts on genes involved in anaerobic metabolism, suggesting that this key trait, and the ability to colonize human intestinal tracts, is a key step in the evolution of MDR clones of E. coli.

## INTRODUCTION

Escherichia coli is now the most common cause of bloodstream infections in the developed world, outnumbering cases of Staphylococcus aureus bacteremia by 2:1 (1). E. coli is also the most common cause of urinary tract infections (UTI), which in turn are among the most common bacterial infections in the world (2). Bacteremia and UTI are caused by a subset of E. coli termed extraintestinal pathogenic E. coli (ExPEC). ExPEC are not a phylogenetically distinct group of E. coli but rather represent strains which have acquired virulence-associated genes that confer the ability to invade and cause disease in extraintestinal sites (3). Genes associated with virulence that confer the ability to adhere to extraintestinal tissues, to sequester extracellular iron, and to evade the nonspecific immune response and toxins resulting in localized tissue destruction have all been described as essential in the process of ExPEC pathogenesis (4).

The problem presented by the scale of ExPEC infections is exacerbated by the number of cases involving multidrug-resistant (MDR) strains (1, 5, 6). Epidemiological surveys report as many as 60% of UTI ExPEC isolates as being resistant to three or more classes of antibiotics and as many as 50% of bacteremia isolates (5, 6). The increase in MDR ExPEC prevalence has been rapid and primarily attributable to a small number of ExPEC lineages (5). The most common of these is the E. coli ST131 lineage, which has rapidly become a dominant cause of ExPEC UTI and bacteremia globally (5–7). E. coli ST131 is particularly associated with carriage of the CTX-M class of extended-spectrum β-lactamase (ESBL) which confers resistance to 3rd-generation cephalosporins (7), and there have been a small number of reports of E. coli ST131 isolates carrying metallo-β-lactamases conferring resistance to carbapenems (8). The carriage of these resistance genes is driven by the acquisition and stable maintenance of large MDR plasmids (9).

The phylogenetic structure of E. coli ST131 is well characterized (10–14) and shows the emergence of a globally disseminated MDR-associated clade C from primarily drug-susceptible clades A and B. The lack of phylogeographic signal and phylogenetic structure based on host source suggests rapid global dispersal and frequent host transitions within clade C (14). Research has suggested that the acquisition of fluoroquinolone resistance via point mutations in DNA gyrase and DNA topoisomerase genes was the primary driver in the rapid emergence of clade C, alongside the predated acquisition of well-defined ExPEC virulence factors (11, 12). Later work also suggested that clade C E. coli ST131 may dominate as a successful MDR clade due to the ability to offset the fitness cost of MDR plasmid acquisition and maintenance via compensatory mutations in gene regulatory regions (14). Genome-wide association studies (GWAS) have been used to identify loci and lineage-specific alleles significantly associated with clade C *E. coli* ST131, which suggested a secondary flagellum locus encoding lateral flagella (Flag-2 [15]), and a number of hypothetical proteins and promoter regions as being clade C E. coli ST131-associated loci (14).

Recent work on E. coli causing bacteremia provided compelling evidence that resistance to antimicrobials has not been the major driver of the success of ST131 (16). An analysis of a large 11-year population survey across the United Kingdom showed that ST131 rapidly stabilized at a level of approximately 20% after its emergence around 2002 in the United Kingdom. This was far in excess of already-resident MDR clones, such as ST88 or ST405. Nevertheless, the overall prevalence of resistance phenotypes remained approximately constant in the population. Furthermore, most currently known major ExPEC clones (primarily ST12, ST73, ST95, and ST69, the last of which also rapidly emerged in 2002) show a similar stable population frequency across the 10 years following the introduction of ST131, despite exhibiting far less extensive resistance profiles. These observations suggested the distribution of ExPEC strains was shaped by negative frequency-dependent selection (NFDS) (16). NFDS describes the situation in which a given phenotype is most beneficial to a population when it is rare, such as the emergence of a new antigen or resource-use strategy. This is because as the phenotype becomes common it either becomes costly, such as when antigens are recognized by an increased proportion of hosts, or less beneficial, such as when strains compete more fiercely for the same resources.

Recently, a multilocus NFDS model of postvaccination Streptococcus pneumoniae population dynamics was described (17). Frequencies of accessory genes were found to be highly conserved across multiple populations on different continents, despite these populations themselves being composed of different strains, as defined by core genome sequences. Detailed modeling and functional analysis indicated changes in strain prevalence could be explained by NFDS driving accessory loci toward equilibrium frequencies, through mechanisms involving interactions with other bacteria, hosts, or mobile elements (17). The levels of the selective force were estimated to be similar across the populations and manifested in the maintenance of stable population frequencies of accessory loci, despite a substantial perturbation of the population by the introduction of the pneumococcal vaccine (17).

Here, we synthesized different genomic analyses to provide a detailed view of the ecology and evolution of ST131. We provide evidence of NFDS shaping the species-wide carried E. coli population using 1,094 systematically sampled bacteremia isolates from the British Society for Antimicrobial Chemotherapy (BSAC) collection. These genomes, and those collated from previous large-scale phylogenomic studies (11–14, 16, 18), allowed a high-resolution analysis of E. coli ST131 using a total of 862 genomes, revealing the steps in the clades’ diversification. This found clade C to have accumulated significantly elevated allelic diversity, particularly enriched for genes involved in anaerobic metabolism and other loci important for colonization of the human host by ExPEC. Our data suggest the evolution of the MDR phenotype is part of a wider ongoing adaptation toward prolonged human colonization that permits expansion despite NFDS due to an adaptive radiation through diversification of metabolic and antigenic loci.

## RESULTS

### Species-wide distribution of the E. coli accessory genome.

The multilocus NFDS model devised to explain the impact of vaccination on the S. pneumoniae population was applied to the 1,094 systematically sampled E. coli isolates from the BSAC collection (16). Collected between 2001 and 2011, this collection revealed that the sequence clusters, or strains, corresponding primarily to sequence types ST69 and ST131 rose rapidly at the start of the sampling period, prior to stabilizing at frequencies of 8% and 18%, respectively. The rise of ST131 was primarily driven by increases in MDR clade C isolates, although isolates from ST69 and ST131 clade B rose in frequency despite them lacking such a resistance profile. It is possible this represents drift in a neutral population. However, this seemed unlikely, given that the invading genotypes rose quickly and displaced some strains, such as ST10, ST14, and ST144, while leaving the common ST73 and ST95 at quite stable frequencies. Alternatively, these strains could each be filling one distinct separate niche. However, these two strains displace multiple other genotypes, suggesting no strict demarcation between niches. Additionally, there was little evidence of any strain being associated with a range of unique traits. Using the previous analysis of gene content with Roary, the 18 strains with at least ten representatives in the population had a mean of 16.7 private genes (range, 1 to 49), defined as those loci present at >95% in one strain and <5% in all others.

However, this does not mean the association between sequence clusters and accessory loci was random. The same Roary analysis identified 8,311 intermediate-frequency genes, present in between 5% and 95% of the overall population analyzed by Kallonen et al. (16) (see Fig. S2 in the supplemental material). Their distribution exhibited a strong association with individual sequence clusters, as defined by an analysis of the core genome (see Materials and Methods), in agreement with recent *k*-mer-based analyses of this population that showed divergence in the core and accessory genome was strongly correlated (19). Hence, as in S. pneumoniae, strains appear to be defined by a characteristic combination of common accessory loci, rather than distinctive private sequences (14, 20). This suggests sequence clusters may be ecologically distinct but with overlapping niches.

10.1128/mBio.00644-19.2 FIG S2 Distribution of accessory loci relative to the E. coli population structure, displayed using Phandango. (A) Core genome phylogeny encompassing 1,509 E. coli genomes from the BSAC and Cambridge University Hospitals collections, as described by Kallonen et al. (16). (B) Population structure analyses. Each column contains one row for each isolate in the phylogeny. The phylogroup information is reproduced from Kallonen et al. (16). The middle three columns show the different levels of clustering identified by hierBAPS. The rightmost column shows the sequence clusters used in this work, inferred from the hierBAPS analysis by identifying the level of clustering that corresponded to a clonal complex when linking isolates sharing identical alleles at five of the seven multilocus sequence typing loci. (C) Distributions of intermediate frequency loci. The 8,311 loci found between 5% and 95% frequency in the overall population are each represented by a column, cells of which are colored blue when the locus is present in the isolate defining the corresponding row. The vertical stripes indicate those loci stably associate with particular sequence clusters. Download FIG S2, PDF file, 14.5 MB. Copyright © 2019 McNally et al. 2019 McNally et al. This content is distributed under the terms of the Creative Commons Attribution 4.0 International license.

**FIG 1:**
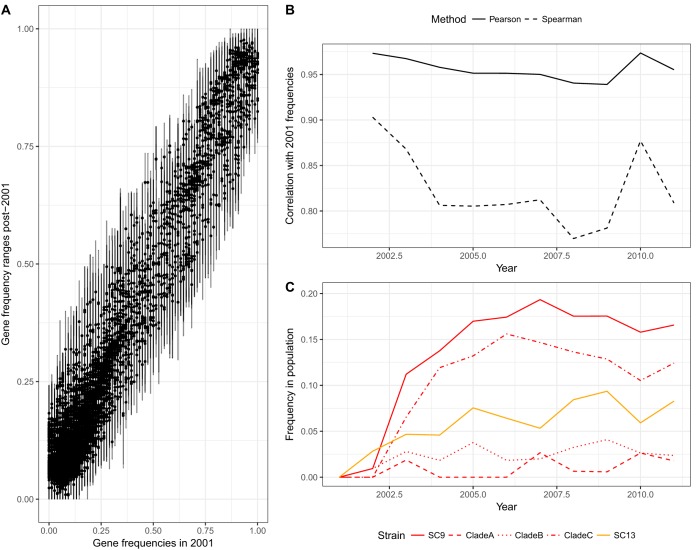
Summarizing the population dynamics of the British Society for Antimicrobial Chemotherapy extraintestinal pathogenic E. coli collection. These isolates were collected from bacteremia cases around the United Kingdom between 2001 and 2011. (A) Conservation of gene frequencies. Each point corresponds to one of the 6,824 genes identified by Roary in the BSAC collection with mean frequencies between 0.05 and 0.95 across all years. Error bars indicate the full range observed across annual samples. (B) Correlation of gene frequencies with those observed in 2001. This shows the changing correlation of gene frequencies, calculated by both the Pearson and Spearman methods, in each year relative to those observed in 2001. Both measures indicate a divergence in gene frequencies as ST69 and ST131 emerge, until 2010, at which point there is a reversion to the frequencies seen in the original population. (C) Emergence of ST69 (SC9, in orange) and ST131 (SC13, red). The frequencies of the subclades of ST131 are shown by the red dashed lines.

We therefore tested whether the population-level frequencies of these intermediate frequency genes were conserved, as following perturbation by vaccination in S. pneumoniae. Comparisons between the pre-ST131 2001 samples and subsequent data from up to 2011 found strong linear correlations between the prevalences of their intermediate-frequency genes (Fig. 1A, see also Fig. S3A). This is consistent with these loci existing at “equilibrium” frequencies, determined by their costs and frequency-dependent benefits. These correlations with the first sample, in 2001, weakened with samples from until 2008, as might be expected with neutral drift (Fig. 1B), as ST131 and ST69 became more prevalent (Fig. 1C). However, the gene frequencies “bounced back” toward their original frequencies in 2008 (21), as indicated by the increased correlation with the 2001 frequencies (Fig. 1C). This elevated correlation was sustained in subsequent years, despite the persistently high frequencies of ST131 or ST69, suggesting a reconfiguration of other lineages in the population.

**FIG 2:**
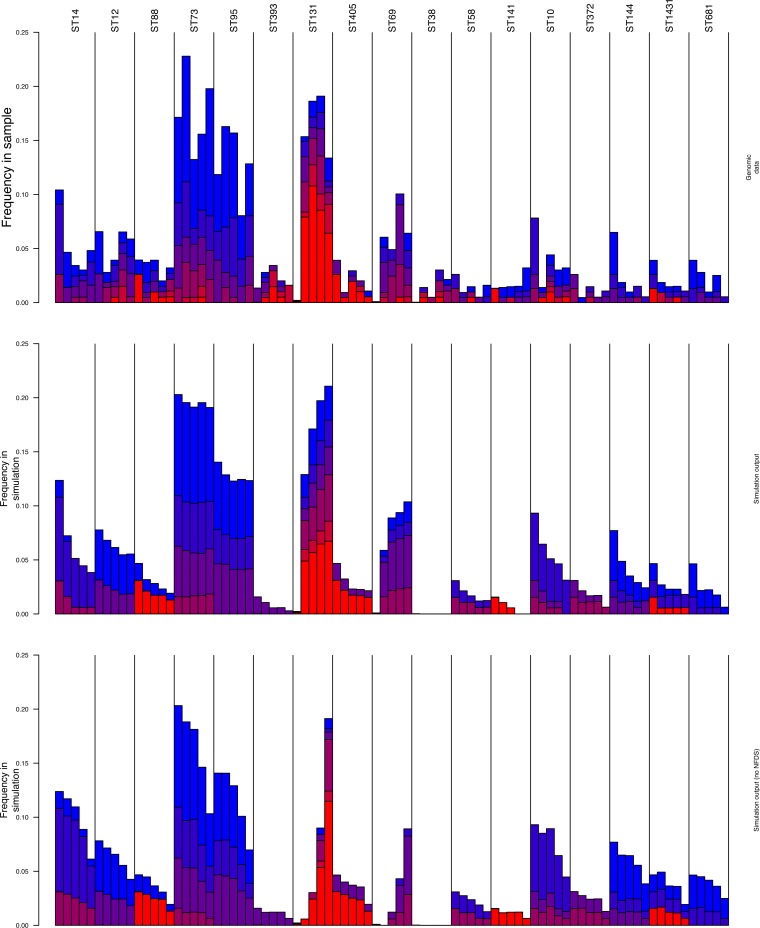
Simulations of changes in the BSAC extraintestinal pathogenic E. coli population evolving under multilocus NFDS. Genomic data (top) and median frequencies (middle) observed from 100 simulations run with the best-matching parameter set identified by fitting the model with BOLFI. This corresponded to σ*_f_* = 0.029, *r *=* *0.179, *m *=* *0.001, *p_f_* = 0.425, and σ*_w_* = 0.0048. Each column corresponds to a sequence cluster identified by hierBAPS (see Materials and Methods) and is annotated with the predominant sequence type with which it is associated. Each bar indicates the frequency of the sequence cluster in consecutive time periods, from left to right. The bars are colored according to the number of antibiotic resistance phenotypes associated with the isolates within the sequence cluster at different time points. (Bottom) The equivalent best fit in the absence of NFDS. Only sequence clusters reaching a frequency of at least 2.5% at one time point in the genomic sample are shown; the full results of the simulation, including measures of between-simulation variation, are shown in Fig. S3.

10.1128/mBio.00644-19.3 FIG S3 (A) Correlations of gene frequencies in the BSAC collection over time. Each plot shows the frequencies of those genes, identified by Roary, that were found to be present at a mean frequency between 0.05 and 0.95 across the entire collection. These graphs show how the correlation between the starting frequencies, in 2001, and those in later years weakened until 2008, at which point the correlation strengthened considerably in 2010 and 2011. (B) Full results of the NFDS simulations. These bar charts show the frequencies for all lineages from the 100 simulations performed using the optimal parameters identified within the BOLFI model fitting, which are summarized in Fig. 2. Each column again corresponds to a sequence cluster and is annotated according to the predominant sequence type. The five bars within each column represent the frequencies of the sequence cluster over subsequent time intervals: either that observed in the genomic samples for the top panel or the median frequency in simulations in the bottom panel. The error bars on the bottom panel indicate the interquartile ranges from the 100 simulations. The red bars correspond to the ST69 and ST131 sequence clusters that had a reproductive fitness benefit, *r*, over the rest of the population. Download FIG S3, PDF file, 0.3 MB. Copyright © 2019 McNally et al. 2019 McNally et al. This content is distributed under the terms of the Creative Commons Attribution 4.0 International license.

### Multilocus NFDS modeling of the E. coli population.

To obtain a population-wide view of these dynamics, the previously described multilocus NFDS model was applied to this data set to test whether these strain dynamics were consistent with selection of the phenotypes encoded by a set of accessory loci, *L*. *L* was primarily composed of the 7,204 genes, identified by Roary, present at intermediate frequencies (between 5% and 95%) in the 2001 sample. These prevalences were assumed to represent the equilibrium frequencies, *e_l_*, determined by NFDS in an unperturbed population (17). As only a single sample was available that predated the emergence of ST69 and ST131, these *e_l_* estimates are likely to be associated with considerable noise; however, the bounce back of accessory locus frequencies toward these *e_l_* suggests they are likely to be accurate for a high proportion of the modeled loci. Additionally, *L* included seven antibiotic resistance phenotypes present in the same intermediate frequency range. Although mechanistic models disagree as to whether antibiotic resistance is under NFDS directly (22) or linked to other loci under balancing selection (23), the stability of individual phenotype frequencies in this population (16) suggests this simplifying assumption is reasonable. These were then simulated as evolving under NFDS; a fraction *p_f_* evolved under strong NFDS, determined by the parameter σ*_f_*, while the rest evolved under weak NFDS, according to parameter σ*_w_* (see Materials and Methods).

The model was initialized with the 2001 population, which was seeded with genotypes observed in later years at a low level, representing the possibility they were present in the population but unsampled. Subsequent simulation with a Wright-Fisher framework included these post-2001 genotypes migrating into the population at a rate *m*. Each isolate was assigned to a sequence cluster, or strain, through identifying clusters in the previously published hierBAPS analysis that approximately corresponded to a multilocus sequence typing clonal complex (see Materials and Methods). The invasion of the resident population by the sequence clusters corresponding to ST131 and ST69 was driven by an increased reproductive fitness relative to the rest of the population, parameterized in the model as *r*. Fitting this model through approximate Bayesian computation (ABC) using BOLFI estimated the parameters listed in Table S2a, which identified significant evidence for NFDS (σ*_f_* and *p_f_* greater than their respective lower bounds used in fitting). The 95% credibility interval σ*_w_* was not higher than the lower bound used in fitting, which might represent a genuine absence of NFDS acting on these loci or a lack of power to detect NFDS in this population over this interval. The conclusion that NFDS acting on at least a subset of accessory loci accounts for the observed population dynamics better than equivalent simulations in which NFDS is negligible provides a quantitative gene-level model that explains the previous strain-level observations of Kallonen et al. (16).

10.1128/mBio.00644-19.9 TABLE S2 (a) Parameter estimates (associated 95% credibility intervals in parentheses) derived from sequential Monte Carlo sampling of the BOLFI model fitted through 500 iterations of model simulation. (b) Tajima’s D measurements for anaerobic metabolism showing 3 or more allelic variants. Download Table S2, PDF file, 0.3 MB. Copyright © 2019 McNally et al. 2019 McNally et al. This content is distributed under the terms of the Creative Commons Attribution 4.0 International license.

These simulations successfully reproduced several aspects of the observed data (Fig. 2 and S3B). Both ST131 and ST69 rapidly spread through the population, before stabilizing at an equilibrium frequency of ∼20%. This does not occur at the expense of the established common clones, such as ST73 and ST95. Instead, in accordance with the genomic data, the displaced sequence clusters include ST10, ST14, ST144, and ST405. These patterns are qualitatively distinct from an equivalent neutral model fit (Fig. 2, bottom). In these simulations lacking NFDS, both ST131 and ST69 are predicted to exponentially increase in frequency rather than rapidly stabilize at a frequency that permits coexistence with the already-resident strains. All other strains commensurately decrease at accelerating rates, proportionate to their original prevalence, meaning the biggest falls are observed for ST73 and ST95, which were actually unperturbed by the invading strains. The greater invasion rate of ST131 relative to ST69 is an artifact of its higher prevalence in the overall data set, meaning it is seeded at a higher level, rather than a true ecological difference.

10.1128/mBio.00644-19.8 TABLE S1 Collection of *E. coli* ST131 genomes. Download Table S1, CSV file, 0.1 MB. Copyright © 2019 McNally et al. 2019 McNally et al. This content is distributed under the terms of the Creative Commons Attribution 4.0 International license.

### Factors affecting the emergence of ST131.

The estimate of the fitness advantage driving the invasion of ST69 and ST131, *r*, is contingent on aspects of the model structure. The absence of population genomic data predating 2001, or from other locations, makes it difficult to model the initial invasion of ST131. Therefore, isolates from 2002 onwards were seeded in the initial population at a frequency of 10^−3^ to replicate the likely scenario in which these genotypes were already present in the United Kingdom or Ireland in 2001 but not captured by the first sample. Increasing the level at which these strains were seeded by 10-fold resulted in a slightly increased final frequency (see Fig. S4). This frequency should represent an approximate upper bound, beyond which it would be unlikely that the strains would have remained unsampled in 2001. Decreasing the seeding level had an almost negligible effect on the simulated dynamics. Hence, the inferred parameters are not markedly sensitive to the seeding level used in the first time step of the simulations.

10.1128/mBio.00644-19.4 FIG S4 (A) Variation in intermediate gene frequencies, present at between 5% and 95% frequency in the 2001 BSAC population, within sequence clusters. This plot compares the output of two different measures of genome content for each sequence cluster represented by more than 10 isolates in the BSAC collection. Each point corresponding to such a grouping is labelled with the most common sequence type among the isolates it encompasses. ST131 clades A, B, and C are also included as separate groupings, and labeled as such. The horizontal axis shows the estimates of alpha from a Heap’s law model fit to a rarefaction curve calculation from the distribution of intermediate frequency loci identified by Roary. In this model, alpha values of <1 are associated with an open pangenome, whereas alpha values >1 represent closed pangenomes. Lower values are associated with a greater diversity of genes per isolate in the grouping. The vertical axis shows the genomic fluidity, corresponding to the Jaccard distance calculated from a sample of pairwise comparisons, also calculated from the intermediate frequency loci: the point shows the mean value, and the error bars show the sample standard deviations. Higher values are associated with greater dissimilarity between each individual pair of isolates. Clade C is relatively close to the origin, along with a few sequence clusters. These appear to represent groupings in which isolates are radiating from a common ancestor: the openness of the pangenomes results from many genes being present at low frequencies, such that each isolate pair individually differs by relatively few loci. (B) Effect of changing the seeding level on simulation results. The top shows the genomic data to which simulations were fitted, and the bottom shows the median outputs from 100 simulations, as in Fig. 2. Download FIG S4, PDF file, 0.1 MB. Copyright © 2019 McNally et al. 2019 McNally et al. This content is distributed under the terms of the Creative Commons Attribution 4.0 International license.

The estimate of *r* was also affected by the simulated population size. To overcome the greater stochasticity inherent to smaller populations, *r* must be higher for ST69 and ST131 for them to consistently rise from their low initial frequencies in the early phases of the simulations. Hence, the same *r* with a 10-fold smaller population than was computationally feasible for fitting (5 × 10^4^) results in lower median frequencies for ST69 and ST131, whereas maintaining *r* with a 20-fold increased population size results in the emerging strains reaching a higher prevalence by the end of the simulations (see Fig. S5A). Hence, as the effective population size of E. coli across the United Kingdom and Ireland is likely to be greater than 5 × 10^4^, the fitness advantage driving these emerging strains to invade is probably lower than the *r* estimated here.

10.1128/mBio.00644-19.5 FIG S5 (A) Effect of changing the carrying capacity on simulation results. The top row shows the genomic data to which simulations were fitted, as in Fig. 2. The rows beneath each show the median outputs from 100 simulations, as in Fig. 2. The second row reproduces the simulation with the best-fitting parameter set and a carrying capacity of 5 × 10^4^. The third and fourth rows show the effect of decreasing the simulated carrying capacity. These demonstrate the final prevalence of ST69 and ST131 increases with higher carrying capacities, but the effect is small over changes of more than 2 orders of magnitude in the simulated population size. (B) Effect of removing NFDS on the emergence of ST69 and ST131. The top row shows the genomic data to which simulations were fitted, as in Fig. 2. The rows beneath each show the median outputs from 100 simulations, as in Fig. 2. The second row reproduces the simulation with the best-fitting parameter set for a model, including NFDS. The third row shows simulations in which *r* is kept at the same level, but there is no NFDS. The fourth row shows the best-fitting parameter set for a neutral model, in which *r* is adjusted to account for the lack of NFDS. Download FIG S5, PDF file, 0.1 MB. Copyright © 2019 McNally et al. 2019 McNally et al. This content is distributed under the terms of the Creative Commons Attribution 4.0 International license.

The emergence driven by *r* is limited by NFDS, which generally constrains the invasion of new clusters of genetically coherent isolates, as the traits with which they are associated have a smaller net benefit as they increase in frequency. Simulations in which ST69 and ST131 have the same fitness benefit, *r*, in the absence of NFDS confirm this advantage would be sufficient to dominate the population in a neutrally evolving population (Fig. S5B).

This inhibition would be expected to most strongly affect lineages with very similar sets of accessory loci. As the MDR isolates of clade C share the most recent common ancestor of the three clades, around 25 years ago (11), their expansion would be expected to be subject to the strongest inhibition by NFDS. Their rapid rise suggests the effects of NFDS may have been ameliorated by the diversification of accessory loci, such that the per locus constraint of the equilibrium frequencies does not limit the strain’s overall prevalence. Extensive variation suggestive of this diversity has previously been noted with the capsules expressed within clade C (23). Therefore, the distributions of intermediate frequency loci within each sequence cluster and the clades of ST131 were analyzed by estimating the overall gene content through a Heap’s law analysis of pangenome curves (24) and through pairwise analysis of gene content similarity by estimating genomic fluidity (25). Comparing the metrics across the sets of isolates revealed that ST131 was one of a small number of clades that had an open pangenome but low genomic fluidity (Fig. S4B). This implies each pair of isolates is similar, reflecting their recent shared ancestry; but, many isolates have undergone different diversifying recombinations at one or more loci present at an intermediate frequency in the wider population. This can account for the overall clade containing many loci in total, despite its recent origin. This is consistent with the adaptive radiation of a successful strain expanding in its population size.

To better understand this diversification and the basis for the high fitness of ST131 represented by *r*, a comprehensive genomic data set encompassing all known ST131 genome sequences was created to understand the unique characteristics of the ST131 lineage, with particular focus on the successful clades B and C.

### Core and accessory genomic structure of the ST131 population.

**FIG 3:**
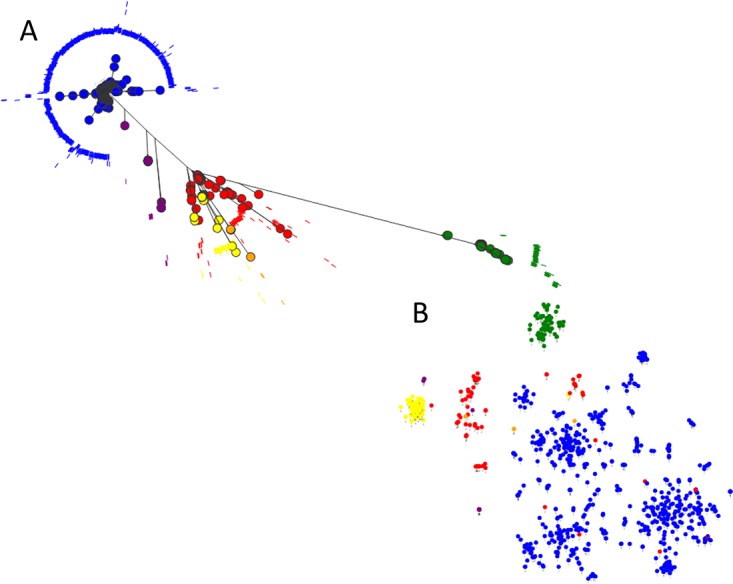
(A) Maximum likelihood phylogeny of 862 E. coli ST131 strains. The phylogeny was inferred using RAxML with a GTR Gamma model of substitution, on an alignment of concatenated core CDS as determined by Roary. (B) PANINI plot of the accessory genome content of all 862 strains based on a tSNE plot. The plot is a diagrammatical representation of the relatedness of each strain based on the presence/absence of accessory genes and is presented as a two dimensional representation. The taxa are color coded by BAPS grouping (Table S1) and show clade A (green, BAPS-3), clade B (red, yellow, and purple, BAPS-2, -4, and -5, respectively), and clade C (blue, BAPS-1).

A maximum likelihood phylogeny generated from an alignment of concatenated core coding DNA sequences (CDS) from all 862 genomes confirmed the earlier consensus three-clade structure of the lineage (Fig. 3A), and in agreement with previous studies, there was no strong phylogeographic signal or host source clustering evident in the phylogeny (https://microreact.org/project/BJKoeBt2b). To confirm that the collation of the 862 genomes was consistent with previous descriptions of the accessory genome distribution in ST131, isolate relatedness based on shared accessory gene content was visualized as a two-dimensional projection using PANINI (Fig. 3B) (26). Clades A and B largely resided in dense clusters at the periphery of the projection. In contrast, clade C isolates were more diffuse, overlapping with some clade B isolates, forming a cloud with discernible substructuring into distinct groups. This concurs with the previous analysis of the gene content of clade C and the previous finding of multiple accessory genome subclusters within this set of isolates (14).

### Low-frequency accessory genes suggest clade A and clade B/C E. coli ST131 rarely cohabit the same ecological spaces.

To identify which aspects of the accessory genome differed between the clades of ST131, the distributions of the 32,631 sets of orthologous genes identified by Roary were analyzed. Characterizing the full set of loci present at intermediate frequencies was not feasible, as even focusing on the 3,354 present at between 5% and 95% frequency revealed that the majority of these were present at a frequency <20% (see Fig. S1). Therefore, the search was refined to clade-specific genes occurring at a frequency of >95% in one clade but at <5% in the other two clades.

10.1128/mBio.00644-19.1 FIG S1 Histograms of the relative frequency of genes within the accessory genome of the entire E. coli ST131 population and of each separate clade. The *x* axes indicate the relative frequencies with which a gene appears, and the y axes indicate the numbers of accessory genes which appear at that given frequency. Download FIG S1, PDF file, 0.3 MB. Copyright © 2019 McNally et al. 2019 McNally et al. This content is distributed under the terms of the Creative Commons Attribution 4.0 International license.

**FIG 4:**
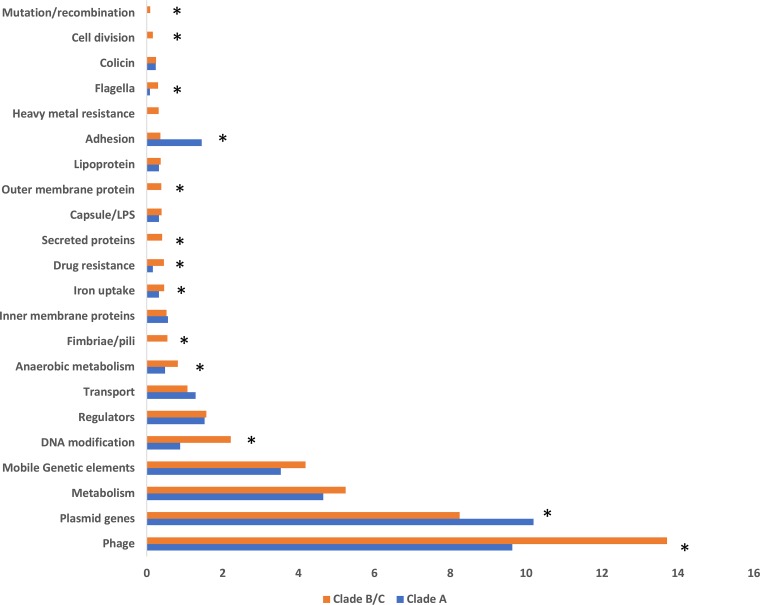
Bar chart depicting functional classes of accessory genes differentially present in clade A and clade B/C E. coli ST131. Functional classes are based on GO classes as described in Materials and Methods. *, significant difference exists between clade A and clade C as determined by *t* test.

In both clade A and clade B/C, the overwhelming majority of low-frequency accessory genes encode hypothetical proteins (64.4% clade A, 58% clade B/C). Excluding the hypothetical proteins from the analysis showed an unexpected bias in functional gene categories differentially observed in the lineages (Fig. 4). The most common gene types were functional phage, plasmid, and other mobile genetic element (MGE) genes, with more private phage genes present in clade B/C than in clade A. Conversely, there were more private plasmid genes in clade A than in clade B/C, despite the presence of a diverse number of MDR plasmids within clade C (14). Together, this suggests that clade A strains of E. coli ST131 and clade B/C strains of E. coli ST131 are exposed to different plasmid and phage pools, an observation which is most parsimoniously explained by them rarely sharing the same ecological habitats at the same time.

Clade A contained the highest number of loci exclusive to a lineage (27) despite constituting the least-sampled clade. This is likely explained by the fact that clade A is on the longest branch of the ST131 phylogeny, and as such, has had most evolutionary time to accumulate differences in the gene repertoire. Clade B had only 2 exclusive loci and clade C had 18. When clades B and C were combined against clade A, there were 60 loci exclusively present in the B/C combination. The majority of clade A private genes encode hypothetical proteins, while those private to clade C encode DNA modification proteins and metabolic functions. The genes private to clade B/C combined also encode hypothetical proteins and metabolic functions, notably, five dehydrogenase enzymes involved in anaerobic metabolism labeled *yihV*, *garR_3*, *fadJ*, *fdhD*, and *gnd* in our data set. A BLAST analysis against the NCBI nonredundant database suggested that the dehydrogenase enzyme gene annotated as *pdxA* in our Roary data set was confined to clade C ST131 strains. These dehydrogenase enzyme genes were found to be present across phylogroup B2 E. coli strains (of which ST131 is a member) through blastn searches of the NCBI nonredundant database. Therefore, these loci are not unique to clade C ST131 and were either acquired by an ancestral clade B/C strain or have been lost by clade A.

### High diversity in core anaerobic metabolism genes unique to clade B/C.

An analysis of accessory loci private to clade B/C (present in >95% of that population) identified two separate loci encoding 3-hydroxyisobutyrate dehydrogenase enzymes and loci encoding 3-hydroxyacyl-coenzyme A (3-hydroxyacyl-CoA) dehydrogenase, 6-phosphogluconate dehydrogenase, and formate dehydrogenase. An analysis of clade B/C loci circulating at a low frequency of <20% also identified a significant overrepresentation of genes encoding dehydrogenase enzymes involved in anaerobic metabolism (a total of 64 loci), including seven variants of formate dehydrogenase. There were also seven variants of the *eutA* gene found in the ethanolamine utilization pathway (the *eut* operon) and a distinct version the *cobW* gene, which encodes the sensor kinase for activation of the cobalamin biosynthesis operon. These anaerobic metabolism genes would normally be considered essential to E. coli. Interrogation of the pangenome matrix for the data set showed that versions of these genes existed as core genome loci. Comparison of the nucleotide sequences of the clade-specific loci with their core genome counterparts showed that these were not genes private to clade B/C *per se* but rather represented multiple unique alleles of genes that are core to the ST131 population, which differ at the nucleotide sequence level by more than 5%. This suggests a unique selection pressure is acting on these core genes in clade B/C compared to those in clade A. The discovery of these core genes that display allelic variation is explained by the fact that the percent nucleotide identity cutoff applied in the formation of our pangenome matrix is 95%, meaning that any alternative alleles of core genes differing by 5% or more will appear in our matrix as accessory genes.

Further scrutiny of low-frequency loci in clade B/C also identified alternative alleles of a large number of well-characterized extraintestinal pathogenic E. coli virulence-associated genes: antigen 43 (7 alternative alleles); heavy metal resistance, such as arsenic (5 loci), copper (4 loci), and mercury (5 loci); capsule biosynthesis (20 loci); cell division and septation (14 loci); antibiotic resistance to chloramphenicol (3 loci), macrolides (2 loci), rifampin (1 locus), and MDR efflux pumps (21 loci); iron acquisition (39 loci); curli and type I fimbriae and P pili (42 loci); lateral and classical flagella (26 loci); and LPS synthesis (9 loci). These variant loci represent alternative alleles of genes found widely across the E. coli phylogeny, indicating there are multiple allelic variants of important genes that are confined to clade B/C of the E. coli ST131 lineage.

**FIG 5:**
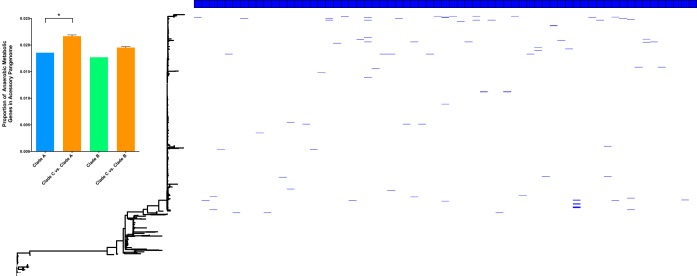
Annotation of a maximum likelihood phylogeny of E. coli ST131, based on concatenated core CDS, with the presence of alternative alleles of 64 loci involved in anaerobic metabolism. Each blue box along the top of the tree annotation represents an individual anaerobic metabolism gene, and its presence in the ST131 population is indicated by a blue line. The inset is a bar chart displaying the proportions of the accessory pangenome that are occupied by genes involved in anaerobic metabolism for ST131 clade A, clade B, subsampled clade C versus clade A, and subsampled clade C versus clade B. *P* = 0.042 for clade C versus clade A and *P* = 0.086 for clade C versus clade B. Error bars represent standard errors of the means. Significance was determined using the median value *P* value from chi-square tests performed on random subsamples of the C clade.

**FIG 6:**
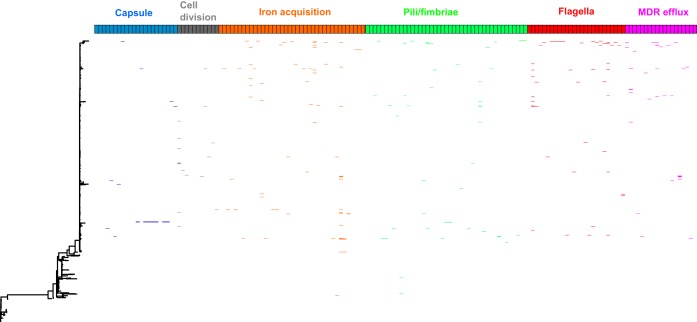
Annotation of a maximum likelihood phylogeny of E. coli ST131, based on concatenated core CDS, with the presence of alternative alleles of loci involved in capsule production, cell division, iron acquisition, pili/fimbriae production, flagella, and MDR efflux pumps. Each box represents an individual gene, and its presence in the ST131 population is indicated by an appropriately colored line.

We sought to determine the distribution of this allelic diversity across the E. coli ST131 phylogeny by annotating the tips of the phylogenetic tree with the presence/absence of each of the anaerobic metabolism (Fig. 5) and capsule, cell division, MDR efflux, iron acquisition, pili, and flagellum divergent loci (Fig. 6). Our analysis shows that each alternative allele occurs at very low frequency, but that alleles are randomly distributed throughout the phylogeny of the C clade and are exclusive to clade C. An analysis of the source attribution of isolates displaying this allelic diversity showed that 80% of isolates displaying allelic diversity were human isolates (32% of those bloodstream isolates, 20% UTI isolates, 2% commensal, and the remainder of an unknown source), 5% were avian isolates, 10% companion animal isolates, and the remainder from other sources. Given that these alleles differ from the normal conserved versions of genes by >5% at the nucleotide level, it is implausible that these alleles would be arising repeatedly and independently via mutation. Instead, the most parsimonious explanation is that the minor frequency alternative alleles are being distributed through the population via recombination. This conclusion is supported by the fact that every one of the allele variants identified in our analysis has 100% nucleotide identity matches with genes present in other E. coli in the NCBI nonredundant database.

Given that our data set is biased toward clade C genomes, we performed comparative analyses of the frequency with which allelic diversity occurs in anaerobic metabolism genes. We randomly subsampled clade C 100 times and compared equal numbers of clade A, B, and C genomes for allelic diversity. Our data show that even when randomly subsampling clade C, the levels of diversity observed in anaerobic metabolism genes is significantly higher than in clade A, providing evidence that the accumulation of sequence diversity is specific to the MDR clade C, despite this clade having the most recent common ancestor of the three (Fig. 5).

Finally, we sought to exclude the possibility that the presence of these allelic variants was skewed by some form of geographically localized expansion of variants. To do this, we compared the relative frequencies of all accessory genes, highlighting the allele variants in anaerobic metabolism, capsule, cell division, MDR efflux, iron acquisition, fimbriae, and flagella present in U.K. versus non-U.K. isolate genomes (see Fig. S6). Our data showed a strong linear relationship between the frequency of genes in the two populations, indicating that the data were not biased by expansion of alleles in a given geographical location, and that this accumulated diversity was equally as likely to happen in any given strain independent of its geographical origin.

10.1128/mBio.00644-19.6 FIG S6 Frequency dependence plot showing the frequency at which all E. coli ST131 accessory genes occur in strains isolated from the United Kingdom versus strains isolated from outside the United Kingdom. The allele variants are color coded as in the other figures: anaerobic metabolism (blue), capsule production (pale blue ), cell division (black), iron acquisition (orange), pili/fimbriae production (green), flagella (red), and MDR efflux pumps (pink). Download FIG S6, JPG file, 0.1 MB. Copyright © 2019 McNally et al. 2019 McNally et al. This content is distributed under the terms of the Creative Commons Attribution 4.0 International license.

### Allelic diversity of anaerobic metabolism genes in clade C ST131 is not observed in other dominant ExPEC lineages.

**FIG 7:**
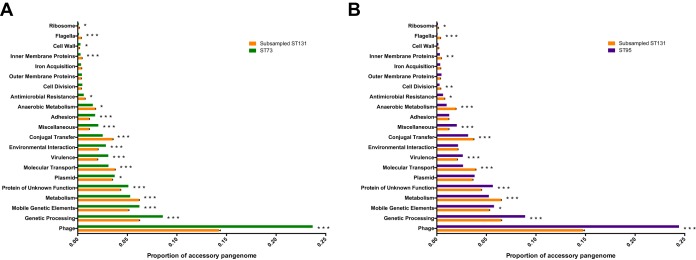
Bar charts depicting the compositions of the accessory genomes of ST73 (A) and ST95 (B) compared to a repetitively sampled clade C ST131. The proportions of the accessory genome are plotted against manually assigned functional categories. Hypothetical proteins are responsible for the majority of the accessory pangenome and are omitted from the graphs. Error bars are standard errors of the means. Iterative chi-square tests were performed to assess significance, as described in Materials and Methods. *, *P* < 0.05; **, *P* < 0.01; ***, *P* < 0.001.

The possibility exists that the above observations made for clade C of E. coli ST131 simply reflect the general evolutionary path of a successful commensal and extraintestinal pathogen. To test this, we performed an identical analysis on the pangenome of 261 ST73 isolates and of 160 ST95 isolates from the U.K. BSAC population survey (16). E. coli ST73 and ST95 represent two of the most dominant lineages associated with clinical extraintestinal disease alongside ST131 (5, 16) but are predominantly non-MDR lineages and rarely associated with MDR plasmids (16). As with our interclade comparisons, we randomly subsampled clade C ST131 100 times to allow equal numbers of genomes per lineage to be compared. Our analysis showed higher levels of plasmid, phage, and hypothetical proteins in the accessory genomes of both ST73 and ST95 than in ST131 (Fig. 7). ST73 and ST95 displayed similar ratios of alternative alleles in adhesins, cell division, and septation genes and multiple iron acquisition genes as observed in ST131 but a higher diversity in virulence-associated genes. However, enrichment in allelic variation in anaerobic metabolism genes was significantly higher in any given subsampled set of clade C ST131 genomes than in both lineages. This supports the hypothesis that the observation of increased diversity accumulating in anaerobic metabolism genes is not a more general extraintestinal pathogenic E. coli trait but is particularly enriched in the ST131 lineage. It also suggests that this is independent of diversification of adhesins or virulence factors, which was enhanced in the ST73 and ST95 lineages.

The accumulation of nucleotide diversity in a given set of loci can often be interpreted as a signature of some form of selection occurring on those genes. However, the low levels of frequency of any given allele across clade C strains contradict a hypothesis for positive selection, where one would expect successful or beneficial alleles to sweep to a high frequency or fixation. Indeed, a comparison of the sequences of each of anaerobic metabolism loci in which three or more alleles were observed identified just three loci which showed a mixture of weakly positive and highly negative Tajima’s D scores, indicating no common selective mechanism in these loci (Table S2b).

However, these results can be reconciled with a lineage evolving under NFDS. Different resource use strategies can facilitate coexistence between competing strains, such those cocolonizing a host, resulting in frequency-dependent selection (28, 29). This would explain the sustained intermediate frequencies of genes encoding dehydrogenases over multiple years (see Fig. S7). Hence, this diversification of metabolic loci could represent the adaptive radiation of particular traits within a successful genetic background, able to efficiently compete with the resident E. coli population through a diverse panel of metabolic capacities suited to exploiting resources under anaerobic conditions.

10.1128/mBio.00644-19.7 FIG S7 Stable intermediate frequencies of anaerobic metabolism loci. Four genes involved in anaerobic metabolism were found to be present at intermediate frequencies in the BSAC collection. All were absent from the ST131 lineage, except nirB_2, which was found in a subset of the lineage. Nevertheless, plotting their annual frequencies reveals distinct stable frequencies over the period, despite the rise to prominence of ST131. Download FIG S7, PDF file, 0.1 MB. Copyright © 2019 McNally et al. 2019 McNally et al. This content is distributed under the terms of the Creative Commons Attribution 4.0 International license.

## DISCUSSION

The evolutionary events that led to the emergence of E. coli ST131 have been an intense focus of research, with consensus opinion suggesting that following the acquisition of key ExPEC virulence factors, the acquisition of fluoroquinolone resistance in the 1980s by the clade C sublineage of ST131 was a key event in that emergence (11, 12). However, a recent nationwide U.K. population survey rejected this hypothesis and suggested that success of the major ExPEC clones is not dictated by resistance traits (16). Here, we combined an analysis of population frequency dynamics with an analysis of the pangenome of the ST131 lineage, strongly suggesting that this species’ population structure and dynamics are shaped by NFDS acting on genomic islands. Such multilocus NFDS is able to account for how an otherwise stable population was disrupted by the invasion of ST131 and ST69, displacing some lineages while leaving other, largely antibiotic-susceptible, genotypes at almost untouched prevalence.

Previous work has suggested that clade C strains of E. coli ST131 undergo reduced levels of detectable core genome recombination compared to those of other phylogroup B2 E. coli (30) or ST131 clade A strains (14). We have previously postulated that this may be a result of ecological separation between clade C strains and other common ExPEC (14, 30). Our analysis of nearly 900 genomes has allowed us to interrogate accessory gene movement to a far greater resolution than previously possible. From the analysis of the accessory genome, we identified thousands of plasmid, phage, and other mobile genetic element genes which are private to clade A and the combined clade B/C. Such an observation is a classic signature of ecological separation of the two populations (31, 32), particularly given that the genetic distance between clade A and clade B/C is much smaller than between other lineages and species from which the circulating genes are also found in the NCBI nonredundant database.

Our analysis also identified a significantly increased level of sequence diversity in genes involved in key host colonization processes in clade C. This diversity was uncovered through our pangenome analysis as allelic variants of core genes. Primary among these is a large number of genes involved in anaerobic metabolism, including seven allelic variants of the formate dehydrogenase gene, as well as allelic variants of genes involved in ethanolamine utilization and cobalamin biosynthesis. The pivotal role of ethanolamine production and cobalamin biosynthesis in the ability of Gram-negative pathogens to outcompete bacteria in the human intestine is well documented (33, 34), and this phenomenon only occurs when supported by an increased ability to perform anaerobic respiration in the presence of inflammation (33). It has been shown that MDR E. coli ST131 is able to colonize the gastrointestinal tract of humans for months or years in the absence of antibiotic selection (35, 36) and that this colonization results in a displacement of the E. coli colonizing the host prior to exposure to the MDR strain (35).

While this diversity in anaerobic metabolism genes was unique to clade C ST131, the allelic variation observed in other human colonization and virulence factors, such as iron acquisition, fimbriae, and cell division, was also observed in two of the other most commonly isolated lineages of E. coli from extraintestinal infections, ST73 and ST95. This diversity likely reflects selection occurring on genes important for ExPEC pathogenesis. Iron acquisition is well characterized as a key virulence determinant in ExPEC, with the ability to initiate successful UTI completely abrogated in the absence of functional iron acquisition systems (37). Recent experimental vaccine work exploiting siderophore production by ExPEC has shown it to be highly effective in rodent models on ExPEC UTI (38). The importance of iron acquisition can also explain many of the MDR efflux allele variants seen in this data set, with half occurring in the *acrD* gene, which has been experimentally shown to play a role in iron acquisition in E. coli (39). We identified multiple alleles of genes in the type 1 fimbria operon and in genes in the P pilus operon, which are classical virulence determinants in UTI (40), and multiple genes involved in capsule biosynthesis, which we have previously reported as being a hot spot for recombination in E. coli ST131 (13, 41). We also identified multiple alleles of genes involved in controlling incomplete septation and filamentous growth, which is a crucial process in the formation of the filamentous intracellular bacterial communities (IBCs) which are thought to be fundamental in the ability of ExPEC to survive inside bladder epithelial cells and cause UTI (42). There are a small number of allelic variants in anaerobic metabolism genes also present in ST73 and ST95, possibly reflecting recent experimental studies suggesting a crucial role for the cytochrome *bd* oxidase system in the ability to cause urinary tract infection (43). Also, previous studies using saturated mutagenesis techniques and studying global transcriptional patterns during urinary tract infection of ExPEC strains have suggested a key role for dehydrogenase enzymes involved in anaerobic metabolism in the ability to cause pathology in the mammalian urinary tract (27, 44, 45).

Recent modeling data on why drug-resistant and drug-susceptible populations of bacteria coexist highlighted that any factors which increase the duration of colonization in a human host will also increase the selective pressure for them to evolve antibiotic resistance (23). Hence, both the success of ST131 in invading the population and the association of many isolates in this lineage with an MDR phenotype would be consistent with the accumulating diversity in anaerobic metabolism loci facilitating enhanced persistence within its host, perhaps through an improved ability to outcompete resident commensal E. coli strains. The fact that this selection is only seen in clade C of ST131 suggests that this occurred around the time of the emergence of the lineage as a human clinical threat (13) alongside the development of fluoroquinolone resistance. Subsequent acquisition of MDR plasmids, and the consequent selection for an ability to offset the fitness costs of long-term MDR plasmid maintenance (14), is likely to have occurred as a result of prolonged exposure to selective antibiotic environments during colonization of humans. Nevertheless, neither anaerobic metabolism genes nor antibiotic resistance loci have swept to fixation in ST131, reflecting their fluctuating but stable prevalence in the broader E. coli population.

This diversification can instead be explained by NFDS, under which these genes are beneficial when rare, because they provide an advantage over cocolonizing strains which will typically lack the same metabolic capacities. However, as these traits become more common as ST131 expands, representatives of this lineage will more commonly encounter one another, therefore necessitating further diversification for different clade C representatives to sustain their advantage over competitors. Similarly, the capsule locus diversification previously observed within clade C, resulting in the capsule synthesis locus corresponding to a “hot spot” of recombination (23), could result from NFDS on variable antigens (46), with the host immune system selecting for a diversity of capsule structures as the dominant type becomes more common following ST131’s emergence (16).

This study presents evidence for two complementary aspects of diversification within the E. coli ST131 lineage. The first is ecological niche separation across a lineage, whereby the different clades of ST131 find themselves competing for similar niches, resulting in them very rarely cohabiting the same habitat. This then results in the formation of distinct subclades within ST131. The second is NFDS across a species within the specific environment of the human gut and the diversification of key genes involved in the colonization of the human gut niche to escape the constraints imposed by NFDS. Further studies are required to fully determine the extent to which niche separation and NFDS are either separate or linked processes. Determining whether loci subject to NFDS are also those that determine niche adaptation will be integral to this process. Understanding the processes that govern the epidemiological dynamics of dominant E. coli lineages, and those of similar pathogens causing bloodstream infections, is critical for addressing the public health threat of antibiotic resistance.

## MATERIALS AND METHODS

### Genome data.

For the NFDS modeling study, the 1,094 isolates from the BSAC resistance surveillance bacteremia collection from the United Kingdom and Ireland between 2001 and 2011 were used. These were described by Kallonen et al. (16).

For the ST131-specific study, a collection of 862 E. coli ST131 genomes (see Table S1 in the supplemental material), of which 684 were previously sequenced as part of phylogenomic investigations of the ST131 lineage (10, 11, 13, 14, 16, 47), was analyzed. An additional 184 ST131 isolates from the BSAC bacteremia resistance surveillance project conducted in the United Kingdom and Ireland between 2001 and 2011 (indicated in Table S1) were included. Together, this collection represents bacteria isolated from invasive disease (bloodstream infections, 504 isolates; 58.5%), human asymptomatic carriage (21 isolates, 2.4%), disease resulting from intestinal carriage (UTI, 163 isolates; 18.9%), and bacteria isolated from a range of veterinary livestock, pets, wild birds, and the wider environment (67 isolates, 7.8%). This provides a data set isolated between 1967 and 2013, providing as broad a view of isolate origin as possible within existing data sets.

In an attempt to avoid any issues arising from different assembly or annotation metrics employed in the previous projects, only raw sequence data were downloaded in fastq format using the previously published accession data. *De novo* assembly was then performed on all the genomes using Velvet (48) and annotation using Prokka (49) as previously described (16). A pangenome of the entire data set was constructed using Roary with 95% identity cutoff (50). A concatenated core CDS alignment was made from the Roary output, and a maximum likelihood phylogenetic tree was constructed from the alignment using RaxML version 8.2.8 (51) and the GTR model with Gamma rate heterogeneity.

For comparative lineage analysis, 264 ST73 genomes and 162 ST95 genomes that were sequenced and fully characterized as part of the U.K. BSAC genome study (16) were utilized.

### Multilocus NFDS modeling.

Multilocus NFDS modeling used genomic data from the previous publication analyzing the population dynamic of bloodstream infection E. coli isolates in the United Kingdom (16). This first required splitting the population into genetically coherent clusters of isolates. This is necessary, as the sequence cluster (or “strain”) frequencies are used for model fitting; therefore, the clusters must be coherent enough to behave reproducibly but large enough to overcome the stochasticity of the model. The previous hierBAPS analysis of the core genome provided three levels of hierarchical clustering (52). The highest-level clusters corresponded to individual common strains in some cases but polyphyletic collections of diverse genotypes in others. Therefore, to ensure the sequence clusters used in the model were of similar levels of diversity, available sequence typing data were used to identify the level of clustering that corresponded to clonal complexes. Each of the highest-level hierBAPS clusters was tested by constructing links between single- and double-locus variants. If the isolates formed a single clonal complex, the cluster was defined as a sequence cluster. Otherwise, the second level of clustering was tested in the same way; if these groups represented a clonal complex, they were used as a sequence cluster. Otherwise, the third most fine-grained level of clustering was used to define a sequence cluster. This identified 56 sequence clusters across the population.

The multilocus NFDS model also requires the definition of a set of *L* loci, each of which is associated with an equilibrium population-wide frequency, *e_l_*. The majority of these correspond to accessory genetic loci, identified as sets of orthologous sequences by a previous Roary analysis (16). Only the 7,204 present at between 5% and 95% frequency in the first sample, from 2001, were included in *L* and therefore modelled as evolving toward *e_l_* under NFDS.

The set of *L* loci was supplemented with seven loci associated with resistance phenotypes if they were present within this frequency range in 2001: amoxicillin, clavulanic acid, ciprofloxacin, cefuroxime, gentamicin, piperacillin-tazobactam, and trimethoprim. The first six of these were directly inferred from the previously published analysis. Trimethoprim was instead inferred from the *sul* and *dfrA* alleles identified by Roary; data from the Cambridge University Hospitals collection (16) was used to train a model constructed with the randomForest R library (https://cran.r-project.org/web/packages/randomForest/), which had 93% accuracy when applied back to the training data set. This was used to infer resistance phenotypes for the BSAC collection.

Analysis used the heterogeneous multilocus NFDS model described previously (17), modified to treat a vaccine cost, *v*, as a fitness advantage, *r*. To simulate the invasion of the resident population by ST131 and ST69, all individuals, *i*, of the two sequence clusters containing ST131 (and related sequence types ST5640, ST5387, ST5494, ST5432, and ST5630) and ST69 (and related sequence type ST106) were assigned the same fitness advantage, *r_i_* = *r*. For all other individuals in the population, *r_i_* = 0. Hence, the function defining the number of progeny, *X_i,t_*, produced by *i* at time *t* was

$$X_{i,t}∼\mathrm{Pois}\left(\left(\frac{\kappa }{N_{t}}\right)\left(1+r_{i}\right)\left(1-m\right)\left({\left(1+\sigma_{f}\right)}^{\pi_{i,t}}+{\left(1+\sigma_{w}\right)}^{\omega_{i,t}}\right)\right)$$

In this formula, density-dependent competition is parameterized by the carrying capacity κ, set at 50,000 to represent a large population that is still computationally feasible, and the total number of cells in the simulated population at *t*, *N_t_*. The strength of NFDS was determined by the parameters *p_f_*, σ*_f_*, and σ*_w_*. As previously, the set of *L* accessory loci and resistance phenotypes were ordered according to the statistic Δ*_l_*:

$${\text{Δ}}_{l}=\frac{{\left(f_{l,t>0}-e_{l}\right)}^{2}}{\left(1-e_{l}\left(1-e_{l}\right)\right)}$$

where *f*_*l,t*>0_ is the mean post-2001 locus frequency. If the *L* loci and phenotypes considered to be under NFDS were ordered by ascending values of Δ*_l_*, then *l_f_* was the highest ranking locus meeting the criterion $\frac{l_{f}}{L}\leq p_{f}$. This determined the strength of NFDS acting on each locus. Therefore, the effect of NFDS on the reproductive fitness of individual *i* depended on which loci were encoded in its genome, as represented by the binary variable *g_i,l_*, and on the deviation of their simulated frequencies at time *t*, *f_l,t_*, from their corresponding equilibrium frequencies:

$$\pi_{i,t}=\overset{l_{f}}{\underset{l=1}{\sum }}g_{i,l}\;\left(e_{l}-f_{l,t}\right)$$

and

$$\omega_{i,t}=\overset{L}{\underset{l=l_{f}\;+1}{\sum }}g_{i,l}\;\left(e_{l}-f_{l,t}\right)$$

These summed deviations served as the exponents for the NFDS terms of the reproductive fitness, with *π_i,t_* and σ*_f_* corresponding to those loci under stronger NFDS, and *ω_i,t_* and σ*_w_* corresponding to those loci under weaker NFDS.

The simulations were initialized with a random selection of κ genotypes from the genomic data, which were biased such that those isolates observed in 2001 were represented at 1,000-fold greater frequency than genotypes collected in later years. This was necessary to “seed” the initial population with ST131 and ST69, to facilitate their expansion in a realistic manner in subsequent years. This represents the likely scenario in which they were present in the sampled region in 2001 but were not observed in the necessarily finite set of sequenced isolates. The parameter *m* represented the rate at which all isolates entered the population through migration; this was biased to import all sequence clusters at the same rate, to avoid any fits in which high rates of migration would artifactually replicate the population observed in the later years of the collection (17).

### Model fitting to genomic data.

As in Corander et al. (17), the simulation model was fitted through approximate Bayesian computation (ABC) using the BOLFI algorithm, which has been shown to accelerate ABC inference 1,000 to 10,000 times without loss of accuracy (53). The prior constraints placed on the parameter values were as follows: the lower bound on all parameters was set to 0.0009; the upper bounds were 0.99 for *r_i_*, 0.2 for *m*, 0.99 for *p_f_*, 0.03 for σ*_f_*, and 0.005 for σ*_w_*. We used 500 iterations of the BOLFI algorithm to minimize the Jensen-Shannon divergence of the sequence cluster frequencies in the genomic data and in the simulations, as ascertained through randomly sampling discrete sets of isolates in accordance with the size and timings of the genomes selected for sequencing from the original collection. Convergence of BOLFI was monitored after every 100 iterations and the approximate likelihood estimate was assessed to have been stabilized after 500 iterations (53). The 95% posterior credible intervals for the parameters were obtained using three generations of sequential Monte Carlo sampling with the same default settings as used in Corander et al. (17). The neutral model was fitted by fixing *p_f_*, σ*_f_*, and σ*_w_* at zero and estimating *r* and *m* through 500 iterations of the BOLFI algorithm, followed by sequential Monte Carlo sampling, as with the full model.

### Accessory genome analysis.

The pangenome matrix from Roary was utilized to investigate the presence of clade-specific loci. The PANINI tool was used with the default setting to visualize the accessory gene-sharing patterns in the population available at https://microreact.org/project/BJKoeBt2b (26). PANINI has been demonstrated to provide efficient complementary visual means to phylogenetic trees to accurately extract both distinct lineages present in a population-wide genomic data set and to highlight clusters within lineages, which are explained by rapidly occurring, homoplasic alterations, such as phage infection. Roary was run on the entire data set using the default 95% sequence identity threshold to cluster genes, allowing the separation of genes based on allelic as well functional differences. Based on a frequency distribution histogram (Fig. S1), a locus as assigned as being clade specific if it occurred at a frequency >95% in one clade and at <5% in the other two clades. Loci identified as clade specific were functionally annotated by performing a tblastn analysis of the nucleotide sequence of the loci against the NCBI nonredundant database.

### Functional categorization of pangenomes.

To assess the functional composition of the accessory pangenome, Gene Ontology (GO) terms were assigned to gene sequences from the pangenome. Briefly, representative sequences from the pan genome of ST131 were mapped to orthologous groups in the bactNOG database using the eggNOG emapper utility (54) Mapping was performed using the diamond search algorithm. The output from eggNOG was filtered to remove orthologous groups with no GO terms. The number of genes mapping to an orthologous group was used as a score for GO term enrichment analysis.

### Comparisons of lineage and clade-specific loci.

To compare lineage pangenomes while accounting for differences in the number of genomes, a sampling approach was utilized. Specifically, a subset with a size equal either to the number of ST73 or ST95 genomes was selected at random from the ST131 clade C, while ensuring a cross representation of the phylogenetic diversity of the clade. The functional enrichment of genes in the subset was quantified and statistically compared to the ST73 or ST95 pangenome using a chi-square test. This process was repeated 100 times to produce 100 *P* values, from which the median *P* value was calculated. Utilizing the same subsampling approach, the pangenome composition of clade C ST131 genomes was compared to both the clade A and clade B pangenomes.

Chi-square statistical tests were performed to assess the significance of the observed differences in functional enrichment. Briefly, with each iteration of the sampling procedure, a chi-square test was performed using the functional proportion of the subsampled pangenome as the observed value and the proportion for ST73 or ST95 as the expected value. This generated 100 *P* values from which one can use the average, maximum, or median to assess significance of the observed differences. In addition, proportional Z statistic tests were also performed to assess the significance of the observed difference. The measurements from the 100 replicates of the subsampling procedure were used to generate an average for the proportions as well as to estimate the variance. The tests were conducted using the proportional measurements from ST73 and ST95 as the “true” means and quantifying how distinct the ST131 subsamples were from these reference values.

### Analysis of diversity observed in genes involved in anaerobic metabolism.

The sequences of 64 anaerobic metabolic genes in which allelic diversity was observed were extracted from individual genomes. The nucleotide sequences were then clustered at 80% identity and 80% length using CD-HIT, which was run using the accurate flag and “word size” of 5 (55). An additional CD-HIT script was used to extract gene sequences for clusters with more than 3 genes, the minimum required by MEGA-CC for analysis. The sequences were then aligned using Muscle with default settings (56). The resulting alignment files were analyzed in MEGA-CC to produce measurements of Tajima’s D (57).

### ST131 clade-specific SNPs.

To visualize the ST131 clades A, B, C, C1, and C2 within the ML tree and the PANINI clustering we identified clade-specific single-nucleotide polymorphisms (SNPs) (Table S1) as previously described (16).

### Data availability.

Accession numbers for the reads used in this study are listed in Table S1 with information of year and place of isolation and the results of the *in silico* PCR for clade-specific SNPs.
