# Supplementary figures and images for: Diversification of Colonization Factors in a Multidrug-Resistant Escherichia coli Lineage Evolving under Negative Frequency-Dependent Selection
Source: mBio. 2019 Apr 23;10(2):e00644-19. doi: 10.1128/mBio.00644-19 (PMC6479005; doi:10.1128/mBio.00644-19)

(A)

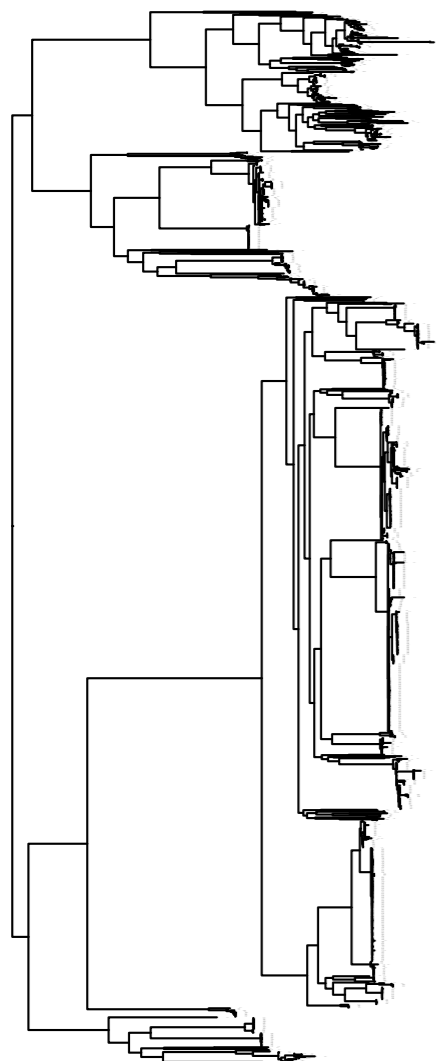

(B)

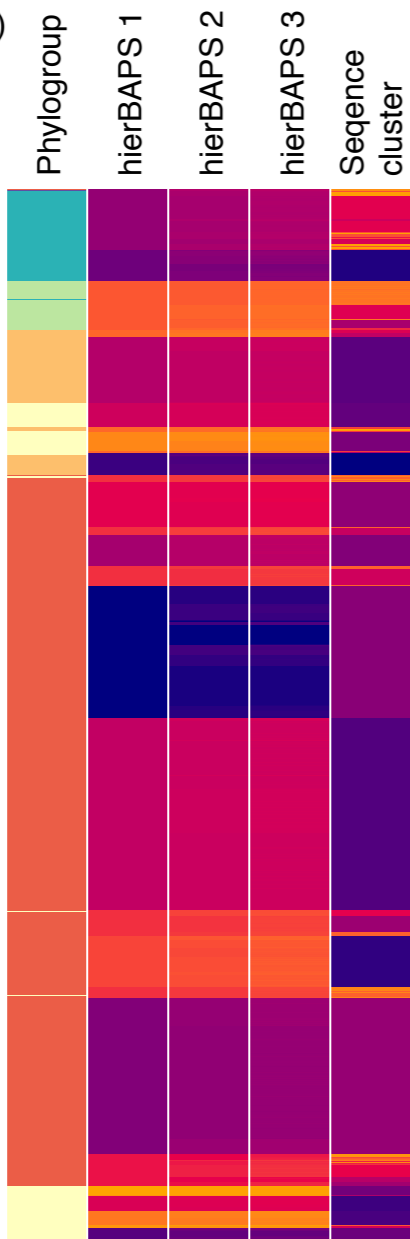

(C)

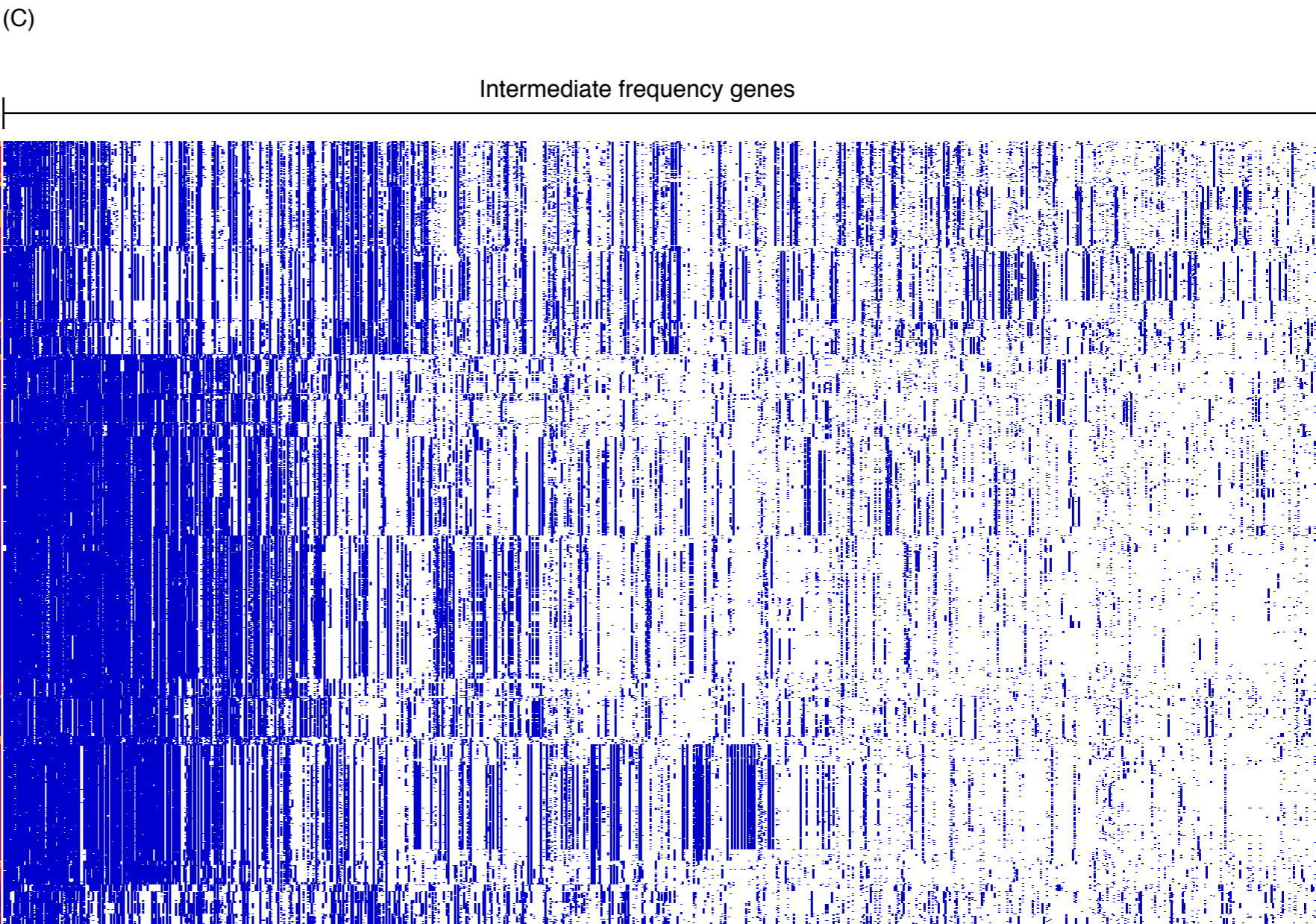

Supplement: FIG S2 [file mBio.00644-19-sf002.pdf]

A

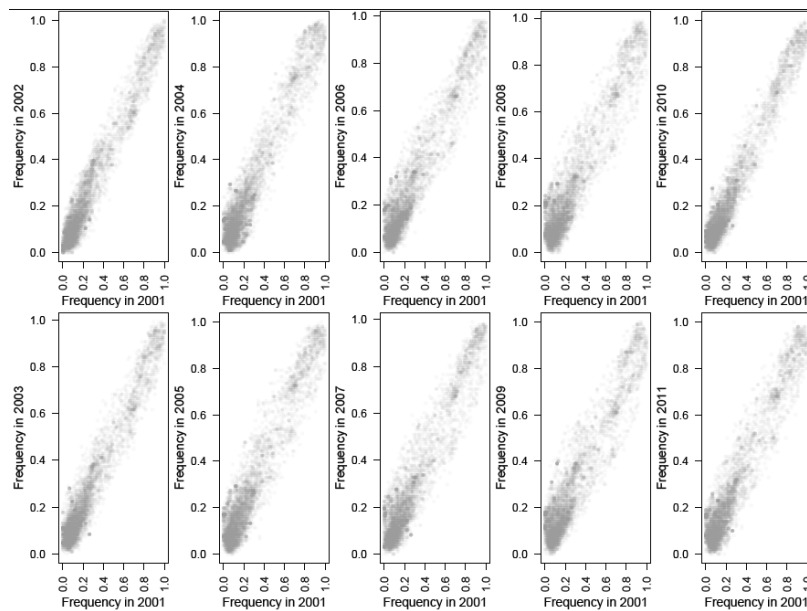

B

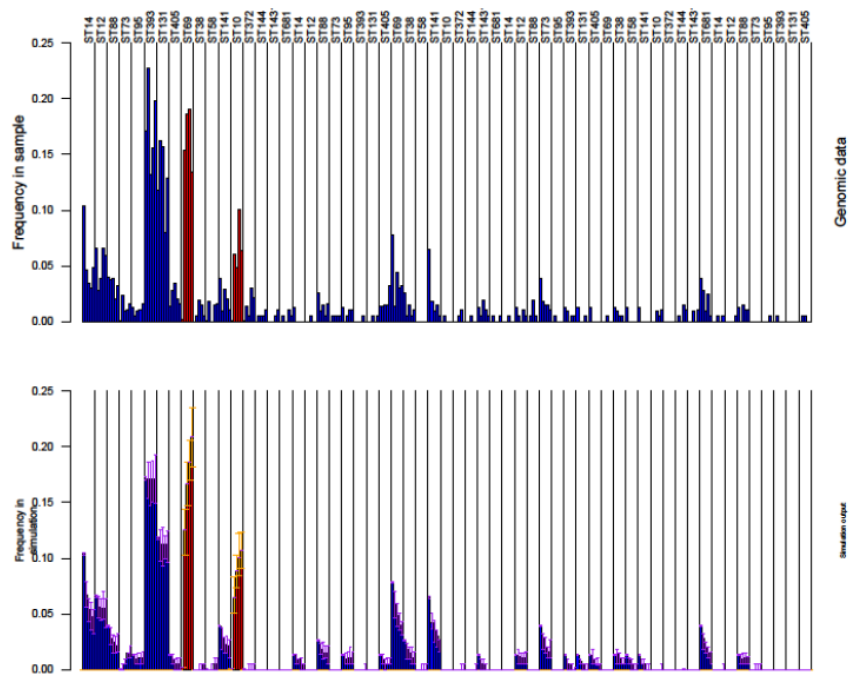

Supplement: FIG S3 [file mBio.00644-19-sf003.pdf]

**A**

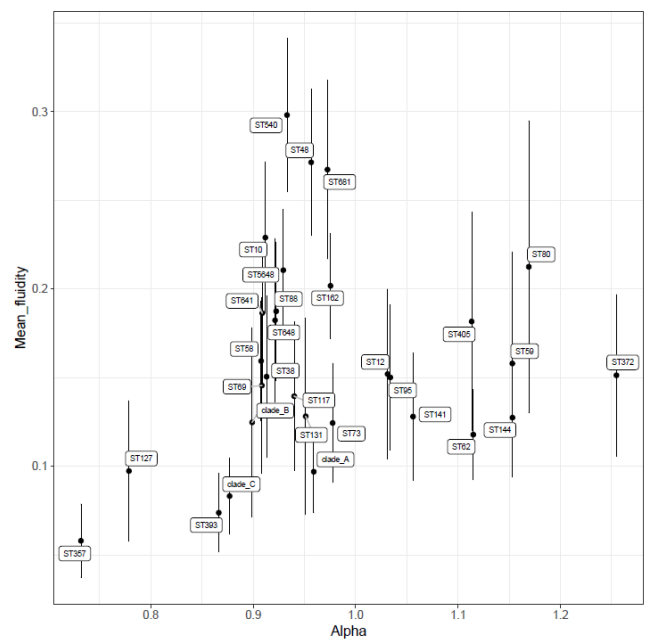

B

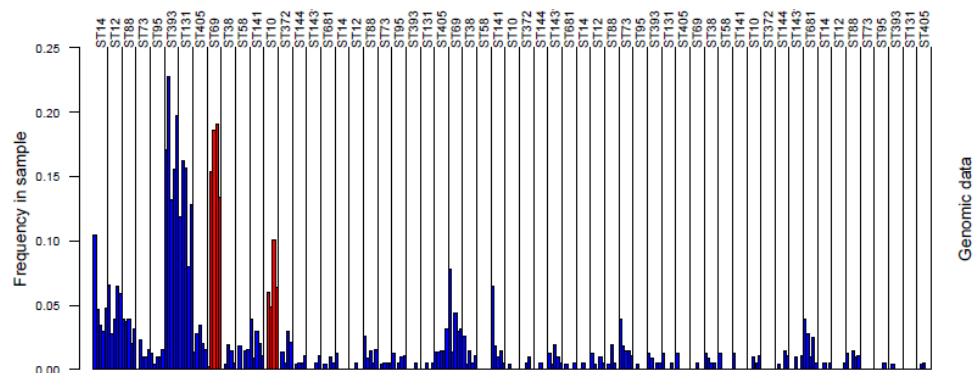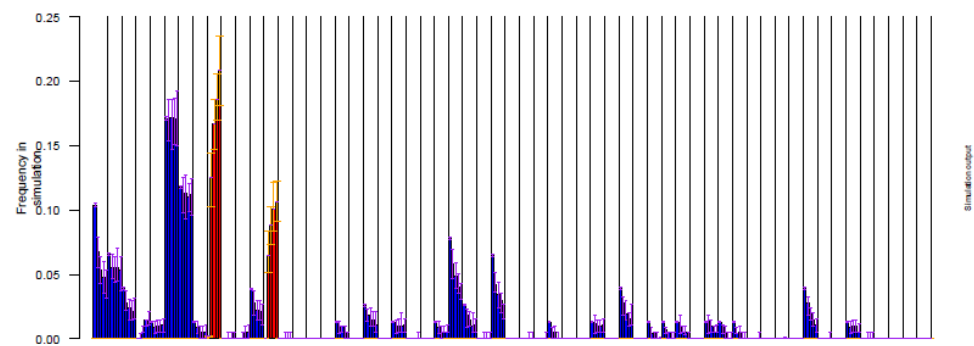

Supplement: FIG S4 [file mBio.00644-19-sf004.pdf]

A

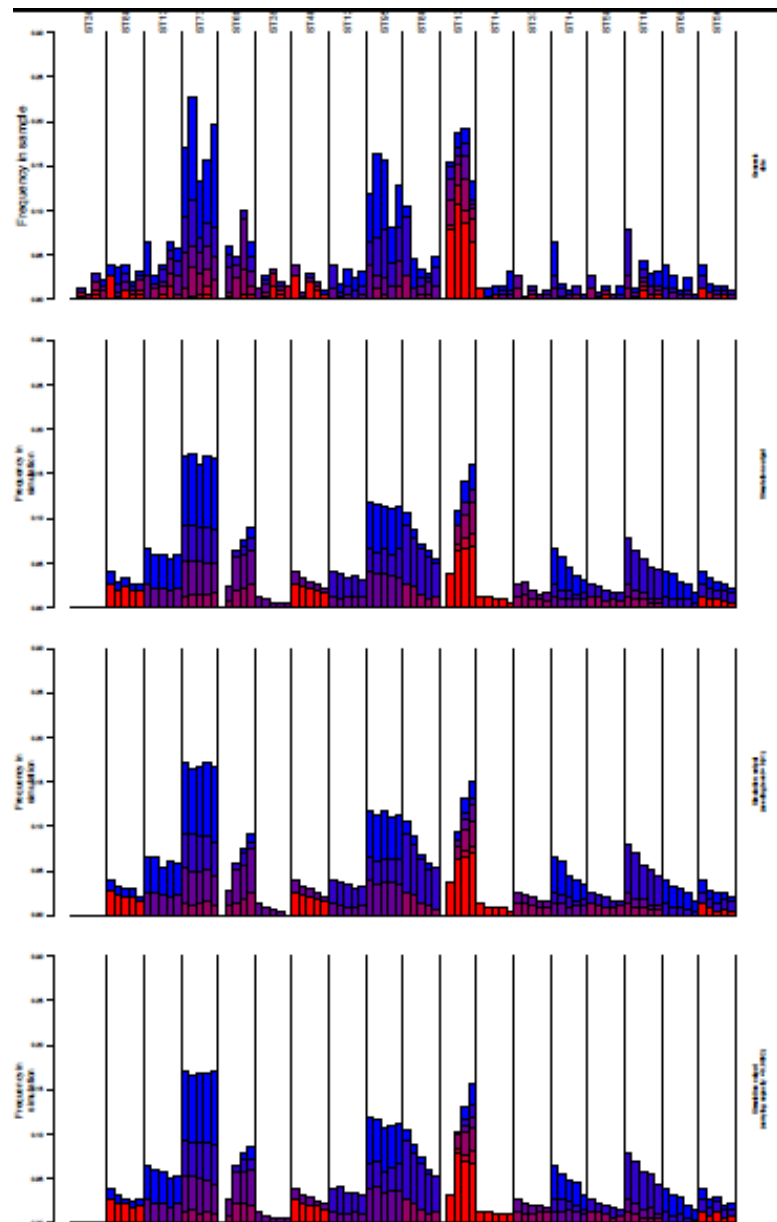

B

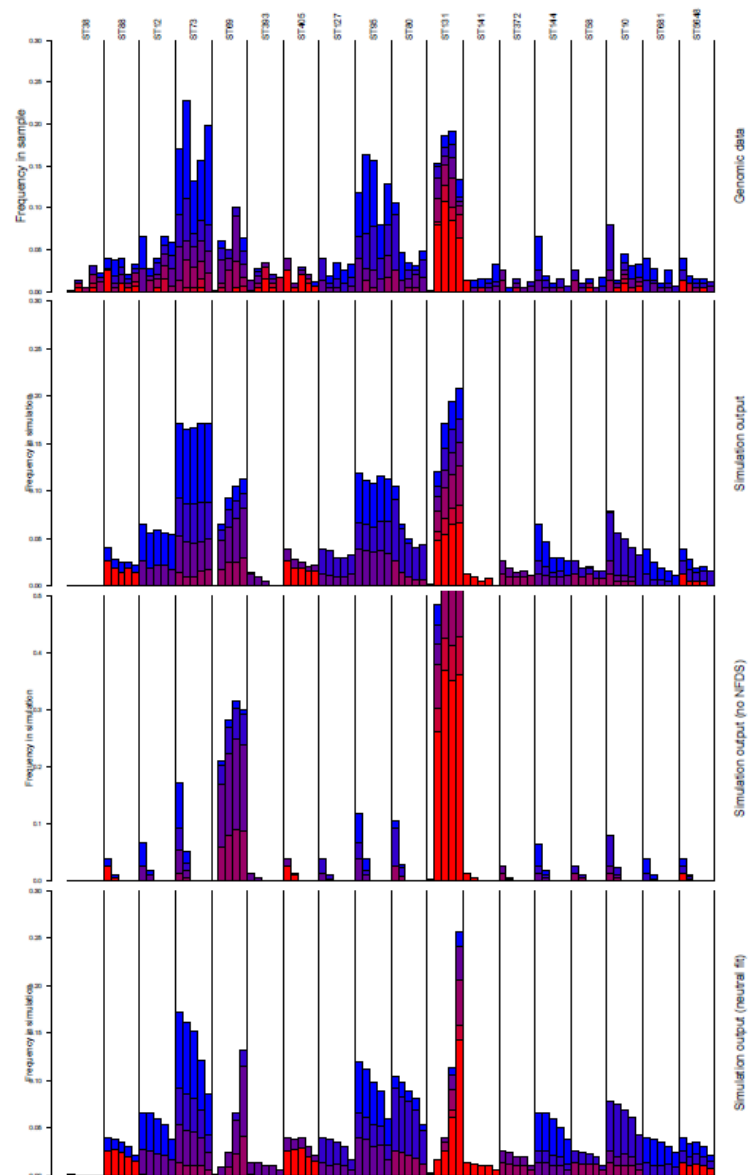

Supplement: FIG S5 [file mBio.00644-19-sf005.pdf]

All

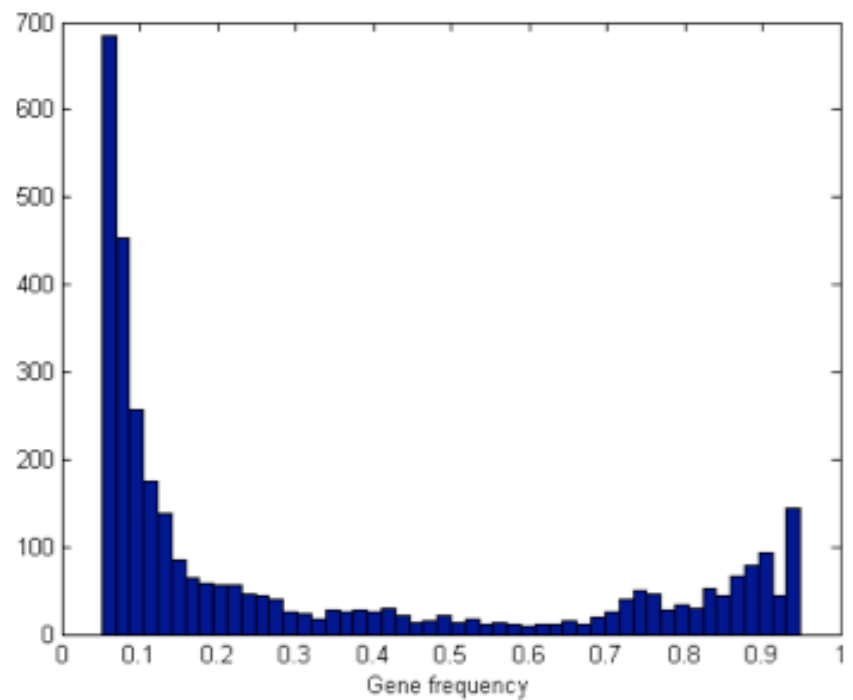

Clade A

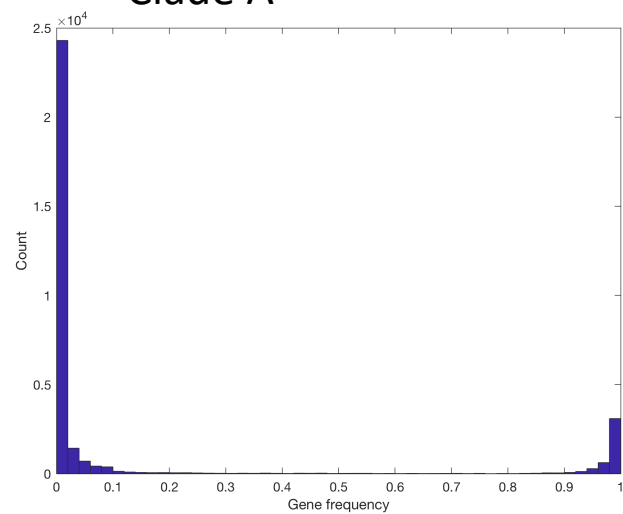

Clade B

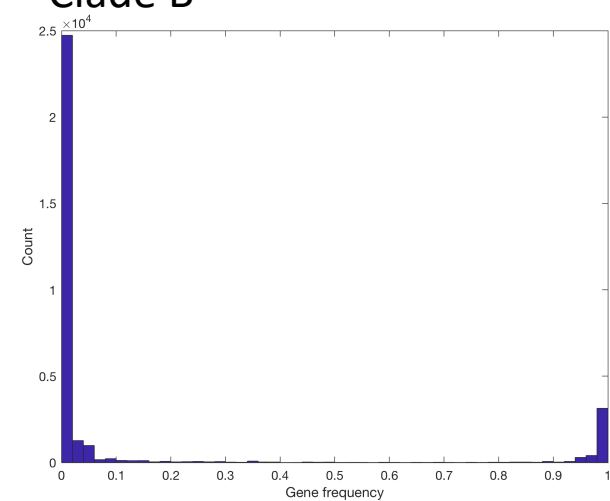

Clade C

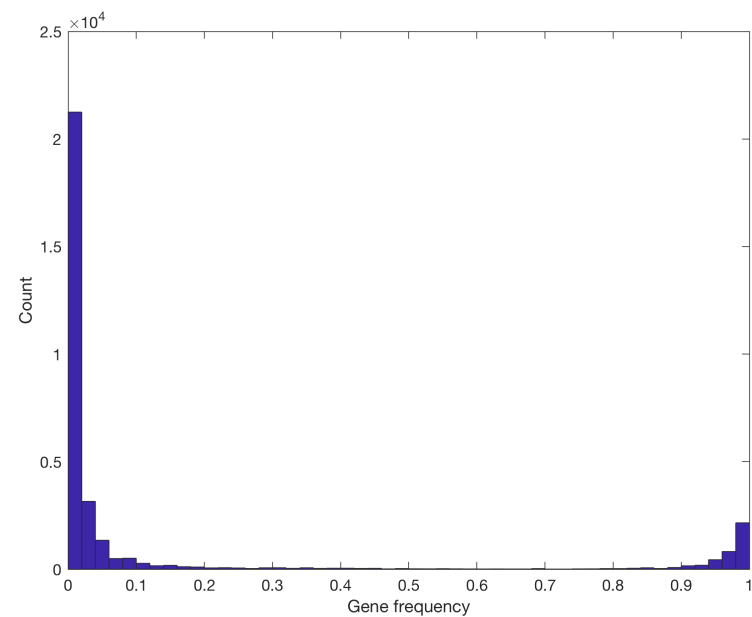

Supplement: FIG S1 [file mBio.00644-19-sf001.pdf]

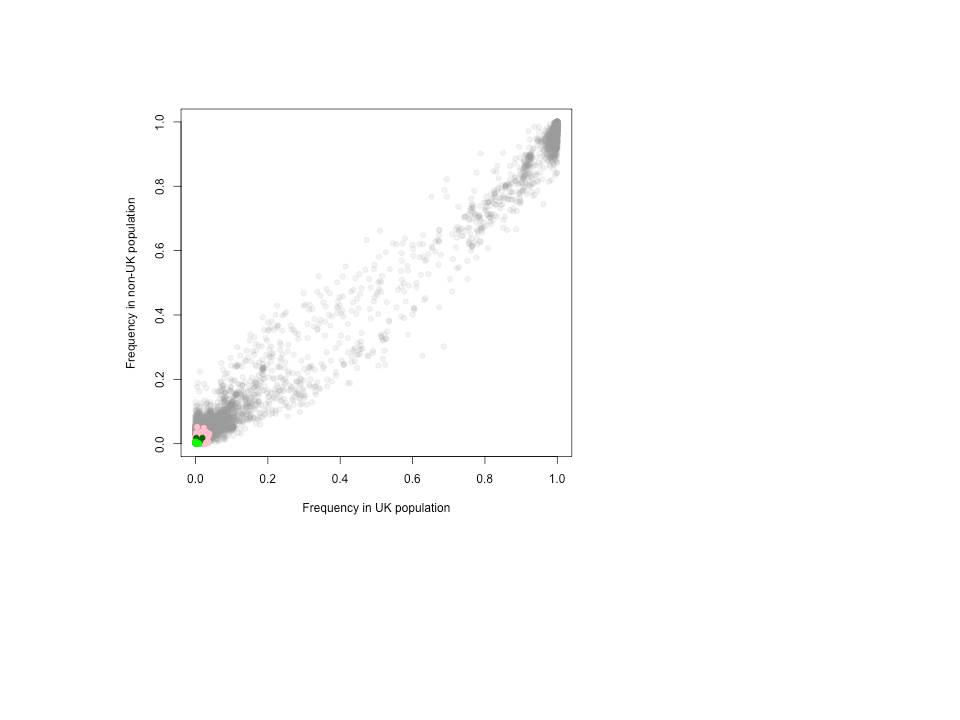

Supplement: FIG S6 [file mBio.00644-19-sf006.jpg]

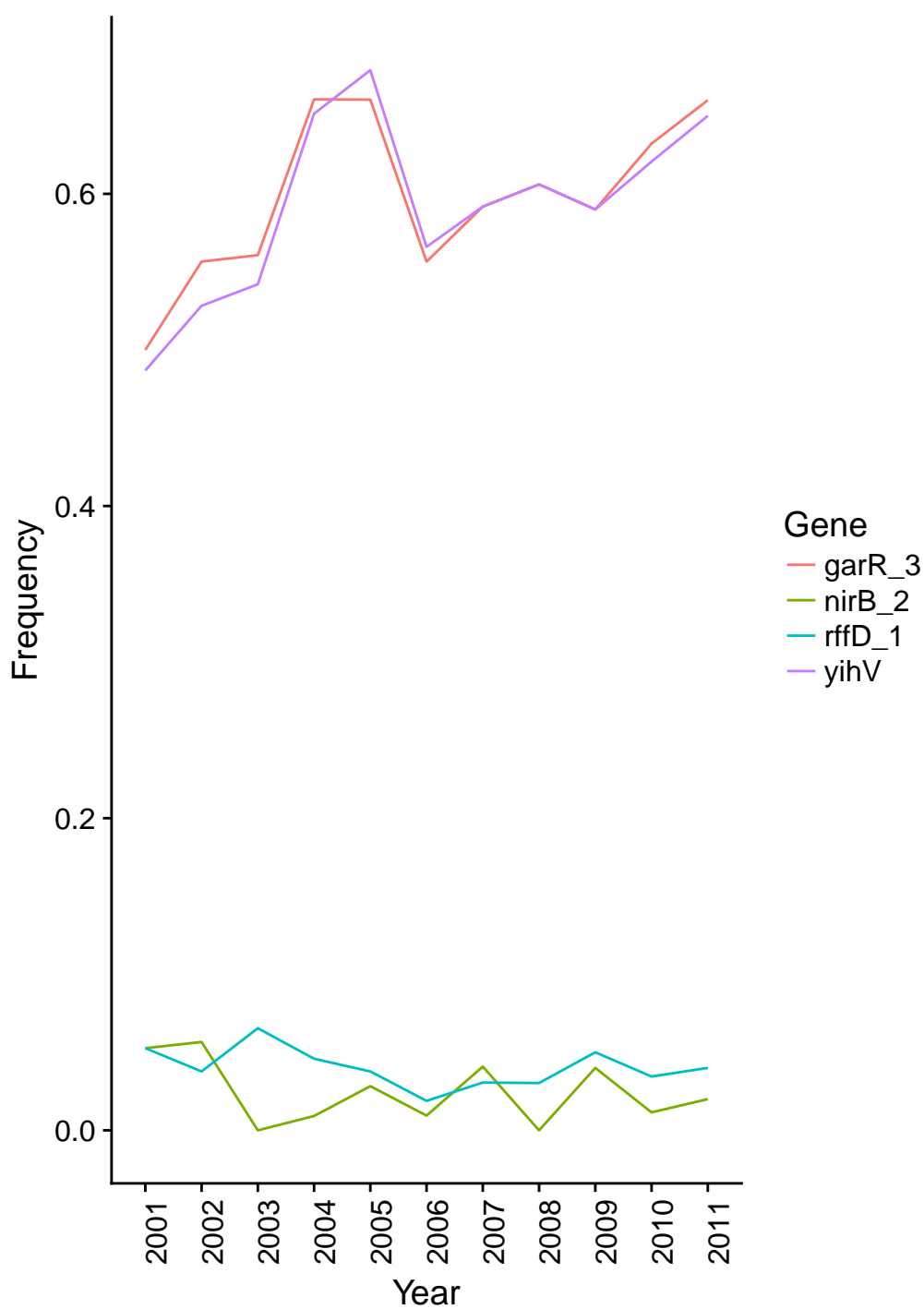

Supplement: FIG S7 [file mBio.00644-19-sf007.pdf]
