# Supplementary material for: Diversification of Colonization Factors in a Multidrug-Resistant Escherichia coli Lineage Evolving under Negative Frequency-Dependent Selection
Source: mBio. 2019 Apr 23;10(2):e00644-19. doi: 10.1128/mBio.00644-19 (PMC6479005; doi:10.1128/mBio.00644-19)
Supplement: TABLE S2 [file mBio.00644-19-st002.pdf]

Table S2a Parameter estimates, and associated 95% credibility intervals in parentheses, derived from sequential Monte Carlo sampling of the BOLFI model fitted through 500 iterations of model simulation. The two fits represent a five parameter fit of heterogeneous NFDS, and a two parameter fit of a neutral model.

**Model fit**	σ_f	σ_w	p_f	r	m
NFDS	0.0128 (0.0027-0.0276)	0.0012 (0.0009-0.0044)	0.4893 (0.1025-0.9773)	0.1527 (0.0917-0.2738)	0.0026 (0.0009-0.0234)
Neutral	-	-	-	0.0474 (0.0374-0.0564)	0.0013 (0.0009-0.0046)

Table S2b Tajima's D measurements for anaerobic metabolism showing 3 or more allelic variants within the ST131 population analysed

Tajima's D Measurements Anaerobic Metabolic Genes

	Gene	Annotated Function	Tajima's D	Number of Variants
1	nirB	Nitrite Reductase [NAD(P)H] large subunit	-1.32e-08	3
2	narY	Respiratory Nitrate Reductase 2 subunit beta	2.840	12
3	eutB	Ethanolamine Ammonia-lyase heavy chain	-0.547	4
4	rffD	UDP-N-Acetyl-D-Mannosamine Dehydrogenase	1.84e-08	3
5	rffE	UDP-N-Acetylglucosamine 2-Epimerase	-6.81e-09	3
6	nemA	Putative NADH:Flavin Oxidoreductase/NADH Oxidase	3.622	20
7	aroD	3-Dehydroquinate Dehydratase	2.350	7
